# Circadian rhythm for female entering old age: exploration on the effect of eating behavior

**DOI:** 10.1093/geroni/igab046.2397

**Published:** 2021-12-17

**Authors:** Hsiao-Han Tang, Ching-Ju Chiu

**Affiliations:** 1 National Cheng Kung University, Institute of Gerontology, College of Medicine, Tainan, Taiwan (Republic of China); 2 National Cheng Kung University, Institute of Gerontology, College of Medicine, National Cheng Kung University, Tainan, Taiwan (Republic of China)

## Abstract

Unhealthy lifestyle and eating behavior are associated with circadian rhythm disruption which contributes to numerous harmful outcomes. The relationship between circadian rhythm and eating behavior remains unclear. The study aims to investigate different types of eating behavior in middle-aged women and their variation in circadian rhythm. A descriptive, cross-sectional design was used. We recruited a convenience sample of 150 female aged 45 years or over from the community in southern Taiwan. Sociodemographic status, sleep diary and eating behavior were collected by questionnaires; behavioral circadian rhythm were monitored with the wrist-worn application. Four middle-aged participants were interviewed. Preliminary data show three main findings: (1) Sleep efficiency was decrease with age, (2) First meal within 2 hours after waking up was associated with higher amplitude (2.24 vs 1.43 log count), relative amplitude (0.92 vs 0.71), middle to vigorous physical activity time (101.22 vs 58.41 minute), lower lowest active 5 hr midpoint (2.63 vs 4.34 hour) and acrophase (13.67 vs 15.75), (3) Participants with morning chronotype have less sedentary behavior and higher most active 10 hr during wake time. Age and timing of first meal after waking up seem dominating circadian rhythm. Chronotype might be a significant factor for physical activity level. More data is needed to further confirm the association.

